# Editorial for the Special Issue on Laser Additive Manufacturing: Design, Materials, Processes, and Applications, 2nd Edition

**DOI:** 10.3390/mi15060787

**Published:** 2024-06-15

**Authors:** Jie Yin, Yang Liu, Linda Ke, Kai Guan

**Affiliations:** 1 Gemological Institute, China University of Geosciences, Wuhan 430074, China; 2 Advanced Manufacturing Research Institute, China University of Geosciences, Wuhan 430074, China; 3 Faculty of Mechanical Engineering & Mechanics, Ningbo University, Ningbo 315211, China; 4 Shanghai Engineering Technology Research Center of Near-Net-Shape Forming for Metallic Materials, Shanghai Spaceflight Precision Machinery Institute, Shanghai 201600, China; 5 TSC Laser Technology Development (Beijing) Co., Ltd., Beijing 100076, China

## 1. Introduction for Special Issue of Laser additive Manufacturing

Laser-based additive manufacturing (LAM) represents one of the most forward-thinking transformations in how we conceive, design, and bring to life engineered solutions. By fusing digital design with material layering processes, LAM transcends the limitations of traditional subtractive manufacturing and equal material manufacturing, enabling the realization of material–micro/macrostructure–performance integration previously considered unattainable [1].

The strategic significance of LAM extends beyond mere production. It embodies a confluence of innovation, sustainability, and efficiency, essential for securing technological advancement and maintaining a competitive edge in the global industrial landscape. With sustainability at its core, LAM minimizes waste through precision use of materials, contributing to a greener manufacturing paradigm [2].

However, to fully harness the power of LAM, we must delve into the intricacies of its many facets. This includes mastering the nuances of material behavior under the influence of laser energy [3,4], optimizing design for additive processes [5,6], understanding the thermodynamics of the laser–material interaction [7], and refining the reliability of the parts produced [8]. Qualified LAM parts must meet stringent requirements, often surpassing those fabricated through traditional means [9].

The pursuit of defect-free, structurally robust parts via LAM is not merely an academic goal but a practical necessity. As we push the boundaries of what can be created, from novel functional devices to high-performance components, we must ensure that the principles of design for additive manufacturing (DfAM) are well integrated with the material science and laser processing techniques that underpin this technology [10]. It is through this synergy that LAM will continue to redefine what is possible in manufacturing, offering a pathway to a new era of industrial capability and innovation.

Building on the work in the first edition [11] and sincere cooperation with the Guest Editors [12], the second edition will continue to concentrate on laser additive manufacturing, including macro- to micro-scale additive manufacturing with lasers, including structure design/material design [13], fabrication [14], modeling and simulation; in situ characterization of additive manufacturing processes [15]; and ex situ material characterization and performances [16], with an overview of various applications [17] in aerospace, biomedicine, optics, transportation, energy, etc.

**Figure 1:**
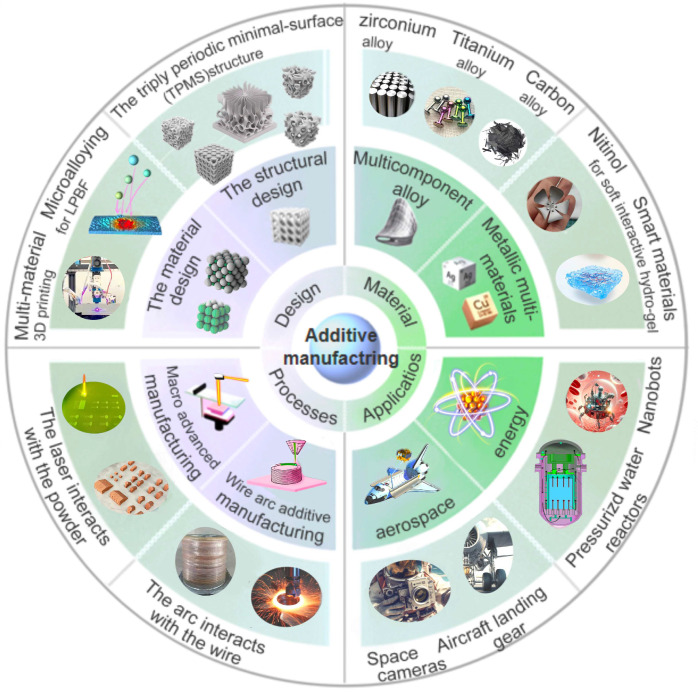
Topics covered in the Special Issue titled “Laser Additive Manufacturing: Design, Materials, Processes, and Applications, 2nd Edition”.

As depicted in Figure 1, this Special Issue featured a diverse array of topics, publishing a total of 11 contributions, comprising 10 original research articles and 1 review paper. After the high-quality reviewing process, four articles [18,19,20,21] were selected as Editor’s Choice. Each of them is briefly introduced below according to four aspects (i.e., design, materials, processes, and applications) of the laser additive manufacturing in this Special Issue.

## 2. Design of Laser Additive Manufacturing in Special Issue

The design of laser additive manufacturing covered in this Special Issue includes the material design (e.g., the high reflectivity and thermal conductivity multi-material 3D printing [22] and the microalloying for LPBF [20]) and the structural design (e.g., the triply periodic minimal-surface (TPMS) structure [18], the thin-walled main support structure [23]).

For the high reflectivity and thermal conductivity multi-material fabrication, Chen et al. [22] reported that the formation mechanism, interface characteristics, and molten pool behavior of the Ag7.5Cu/Cu10Sn (A/C) and Cu10Sn/Ag7.5Cu (C/A) interfaces were printed by laser powder bed fusion (LPBF) for the first time to reveal the influence of different building strategies. They found that the high thermal conductivity of the substrate enhanced the Marangoni convection, which strengthened interfacial bonding strength and reduced the defects.

For the particle-reinforced titanium matrix composites (PRTMCs), Li et al. [20] studied the impact of LaB6 content on the microstructure, quasi-static characteristics, and dynamic properties of titanium matrix composites, focusing primarily on the TC4 and TC4/LaB6 composites manufactured using LPBF. The sample with 0.5 wt.% LaB6 was found to have the best strength–toughness synergy among the three groups of composites due to having the smallest grain size. This study was able to provide a theoretical basis for an in-depth understanding of the compressive properties of additive manufacturing of PRTMCs under high-speed loading conditions.

For the TPMS structure high-precision fabrication, Qu et al. [18] reported the critical roles of energy density and minimal feature size on superelastic NiTi functional components by high-precision LPBF. In this work, extensive parameter studies were conducted to reveal their influences on the microstructure and mechanical properties of as-printed components with different minimal feature sizes (thin wall structure, bulk samples, TPMS structures, etc.). TPMS structures were proposed and verified to be a good candidate as the standard test for parameter study of printing NiTi intricate components and investigation of NiTi thin wall structure mechanical property. Furthermore, a robotic cannula tip with fine minimal feature size and high superelasticity was fabricated using HP-LPBF as a case study.

## 3. Materials of Laser Additive Manufacturing in Special Issue

This Special Issue covers a wide range of forming materials, including zirconium alloys [24], titanium alloys [20,23], steel [21,25,26], composites (e.g., electroless copper and carbon fiber [27]), and in particular, metallic multi-materials (e.g., copper/silver [22]), 4D printing materials (e.g., NiTi, smart materials for soft interactive hydrogel [19]).

Lei et al. [21] reported that AerMet100 steel was produced using an in situ rolling hybrid with wire arc additive manufacturing. In their work, the microstructure, tensile properties, and fracture toughness of as-deposited and heat-treated AerMet100 steel were evaluated in different directions. The results reveal that the manufacturing process leads to grain fragmentation and obvious microstructural refinement of the AerMet100 steel and weakens the anisotropy of the mechanical properties. After heat treatment, the microstructure of the AerMet100 steel is mainly composed of lath martensite and reversed austenite. Alloy carbides are precipitated within the martensite matrix, and a high density of dislocations is the primary strengthening mechanism. The existence of film-like austenite among the martensite matrix enhances the toughness of AerMet100 steel, which coordinates stress distribution and restrains crack propagation, resulting in an excellent balance between strength and toughness. The AerMet100 steel with in situ rolling is isotropy and achieves the following values: an average ultimate strength of 1747.7 ± 16.3 MPa, yield strength of 1615 ± 40.6 MPa, elongation of 8.3 ± 0.2% in the deposition direction, and corresponding values in the building direction are 1821.3 ± 22.1 MPa, 1624 ± 84.5 MPa, and 7.6 ± 1.7%.

Wang et al. [27] investigated the effects of building directions on the crystallographic anisotropy, phase composition, superelastic properties, microhardness, geometrically necessary dislocation (GND) density, and impurity element content of NiTi shape memory alloys (SMAs) fabricated by LPBF. The main idea of this study was to change the introduction of impurity elements in NiTi alloys and alter their microstructures by adjusting the LPBF building direction, which in turn affects their phase transformation temperatures and superelasticity. In conclusion, for engineering applications, the 45° sample had a lower phase transformation temperature, which was more favorable for superelastic recovery under certain temperatures (austenite start temperature (As) < testing temperature < austenite finish temperature (Af)), while the preferred orientation of the 90° sample ensured a greater superelastic recovery strain when tested at Af + 10 °C.

Wang et al. [28] use a nanosecond ultraviolet laser to study the effects of laser process parameters on the adhesion strength between electroless copper and CFCs. To achieve good adhesion strength, four key process parameters, namely, the laser power, scanning line interval, scanning speed, and pulse frequency, were optimized experimentally using response surface methodology, and a central composite design was utilized to design the experiments. The numerical analysis indicated that the optimized laser power, scanning line interval, scanning speed, and pulse frequency were 5.5 W, 48.2 μm, 834.0 mm/s, and 69.5 kHz, respectively. A validation test confirmed that the predicted results were consistent with the actual values; thus, the developed mathematical model can adequately predict responses within the limits of the laser process parameters being used.

## 4. Processes of Laser Additive Manufacturing in Special Issue

This Special Issue focuses on macro-advanced manufacturing (e.g., laser powder bed fusion (L-PBF) [18], wire arc additive manufacturing (WAAM) [21]), but also on micro-advanced manufacturing (e.g., laser photochemical synthesis [19]). Furthermore, the process optimizations on the adhesion strength [28] and the mechanical properties [24] (in-process) and the influence of aging treatment regimens on the microstructure and mechanical properties [25] (post-process) were investigated.

Song et al. [24] reported the influences of process parameters on the printability, surface roughness, and mechanical properties of the LPBFed nuclear Zr-4 alloy. The results showed that the relative density of the Zr-4 alloy samples was greater than 99.3% with the laser power range of 120–160 W and the scanning speed range of 600–1000 mm/s. Under a moderate laser power in the range of 120–140 W, the printed Zr-4 alloy possessed excellent surface molding quality with a surface roughness less than 10 µm. The microstructure of the printed Zr-4 alloy was an acicular α phase with an average grain size of about 1 µm. The Zr-4 alloy printed with a laser power of 130 W and a scanning speed of 400 mm/s exhibited the highest compression strength of 1980 MPa and the highest compression strain of 28%. The findings demonstrate the potential in the fabrication of complex Zr-4 alloy parts by LPBF for industrial applications.

Liu et al. [26] investigated the surface morphologies and corrosion behaviors of LPBFed 316L stainless steel polished with different laser pulse widths. The experimental results show that, compared to the nanosecond (NS) and femtosecond (FS) lasers, the surface material’s sufficient remelting realized by the continuous wave (CW) laser results in a significant improvement in roughness. The surface hardness is increased, and the corrosion resistance is the best. The microcracks on the NS laser-polished surface led to a decrease in the microhardness and corrosion resistance. The FS laser does not significantly improve surface roughness. The ultrafast laser-induced micro-nanostructures increase the contact area of the electrochemical reaction, resulting in a decrease in corrosion resistance.

Dong et al. [25] studied the effects of aging temperature and time on the microstructure and mechanical properties of SLM 17-4 PH steel. They found that the aging treatment induced the formation of the austenite phase with a face-centered cubic (FCC) structure. With prolonged aging treatment, the volume fraction of the austenite phase increased, which agreed with the EBSD phase mappings. The ultimate tensile strength (UTS) and yield strength gradually increased with increasing aging times at 482 °C. The UTS reached its peak value after aging for 3 h at 482 °C, which was similar to the trend of microhardness (i.e., UTS = 1353.4 MPa). However, the ductility of the SLM 17-4 PH steel decreased rapidly after aging treatment. This work reveals the influence of heat treatment on SLM 17-4 steel and proposes an optimal heat-treatment regime for the SLM high-performance steels.

## 5. Applications of Laser Additive Manufacturing in Special Issue

The findings of this Special Issue are expected to provide guidance and reference for scientists and engineers in the fields of aerospace (e.g., space camera and aircraft landing gear [23]), energy (e.g., fuel envelope positioning lattices of pressurized water reactors, component boxes, and heat exchangers [24]), and micromachines (e.g., four-dimensional microrobots/nanorobots [19]).

Tao et al. [19] reviewed the literature on the four-dimensional micro/nanorobots via laser photochemical synthesis towards the molecular scale. Miniaturized four-dimensional (4D) micro/nanorobots denote a forerunning technique associated with interdisciplinary applications, such as in embeddable labs-on-chip, metamaterials, tissue engineering, cell manipulation, and tiny robotics. With emerging smart interactive materials, static micro/nanoscale architectures have upgraded to the fourth dimension, evincing time-dependent shape/property mutation. Molecular-level 4D robotics promises complex sensing, self-adaptation, transformation, and responsiveness to stimuli for highly valued functionalities. To precisely control 4D behaviors, current–laser-induced photochemical additive manufacturing, such as digital light projection, stereolithography, and two-photon polymerization, is pursuing high-freeform shape-reconfigurable capacities and high-resolution spatiotemporal programming strategies, which challenge multi-field sciences while offering new opportunities. Herein, this review summarizes the recent development of micro/nano 4D laser photochemical manufacturing, incorporating active materials and shape-programming strategies to provide an envisioning of these miniaturized 4D micro/nanorobots. A comparison with other chemically/physically fabricated micro/nanorobots further explains the advantages and potential usage of laser-synthesized micro/nanorobots.

Peng et al. [23] reported the design and optimization of a thin-walled main support structure for a space camera based on additive manufacturing. They proposed a solution for designing and optimizing a large-scale complex thin-walled structure using additive manufacturing. Firstly, we devise an integrated thin-walled structure and test material for the main support. Secondly, shape optimization is achieved via the optimization of the lateral slope angle of the primary support based on Timoshenko cantilever beam theory. Additionally, an active fitting optimization algorithm is proposed for the purpose of refining the wall thickness of the thin-walled structure. Then, we determine the structural design of the main support. This primary support is manufactured via selective laser melting (SLM). Following processing, the structure size is 538 mm × 400 mm × 384 mm, and the mass is 7.78 kg. Finally, frequency scanning experiments indicate that, in the horizontal direction, there is a natural frequency of 105.97 Hz with an error rate of approximately 3% compared to finite element analysis results. This research confirms that our large-scale complex, thin-walled main support structure design meets all design requirements.

To conclude, we would like to acknowledge all the authors for their contributions to the success of this Special Issue in *Micromachines*, as well as the reviewers whose feedback helped to improve the quality of the published papers.
